# Overcoming barriers to use of child car seats in an urban Aboriginal community—formative evaluation of a program for Aboriginal Community Controlled Health Services

**DOI:** 10.1186/s40814-018-0351-z

**Published:** 2018-10-20

**Authors:** Lisa Keay, Kate Hunter, Martyn Ralph, Bobby Porykali, Marilyn Lyford, Kathleen Clapham, Winston Lo, Rebecca Ivers

**Affiliations:** 1 Injury Division, The George Institute for Global Health, University of New South Wales, Sydney, Level 5, 1 King Street Newtown, Sydney, 2042 Australia; 2 The Poche Centre for Indigenous Health, The University of Sydney, Room 224, Edward Ford Building (A27), Sydney, Australia; 3 Cardiovascular Division, The George Institute for Global Health, University of New South Wales, Sydney, Level 5, 1 King Street Newtown, Sydney, 2042 Australia; 4 Australian Health Services Research Institute, University of Wollongong, Building 234 (iC Enterprise 1) Innovation Campus, Wollongong, NSW 2522 Australia; 5 School of Public Health and Community Medicine, University of New South Wales, Sydney, Samuels Avenue, Kensington, Sydney, 2033 Australia

**Keywords:** Child car seats, Booster seat, Aboriginal, Process evaluation, Research methods

## Abstract

**Background:**

Little is known about the barriers to use of child car seats in Australian Aboriginal communities, or the acceptability of programs to increase appropriate car seat use. This formative evaluation sought to consult and partner with Aboriginal Community Controlled Health Services (ACCHS) to develop and evaluate the feasibility and acceptability of a program intended to improve optimal use of child car seats.

**Methods:**

Focus groups were conducted with parents and carers of Aboriginal children to identify the barriers and facilitating factors for child car seat use, and staff of two ACCHS were interviewed to inform program development. Following the implementation of the resulting multi-faceted program, consisting of staff training, education, hands-on demonstrations and a subsidised car seat distribution scheme, interviews were conducted to assess process issues and acceptability with 13 staff members.

**Results:**

Parents and carers in the focus groups reported a lack of awareness of child car seat use, confusion about the right car seats for different aged children but agreed about the importance of safety and community responsibility to keep children safe in cars. Interviews with service staff informed an approach to deliver relevant information. Information and resources were delivered to families, while the car seat distribution scheme supplied 33 families with child car seats. Following the conclusion of the program, staff reported that the program was relevant to their role. They also valued the car seat distribution scheme. Staff training in selection and installation of car seats increased confidence in staff knowledge.

**Conclusions:**

We developed a program to promote child car seat use in ACCHS, which focused on developing capacity, made use of existing infrastructure and developed resources for use in this setting. The program shows promise as a means to promote child car seat use in Aboriginal communities; however, the impact on child car seat use will need to be evaluated in a larger scale prospective trial.

## Background

Road traffic injuries are the leading cause of death and serious injury in Australian children. In 2016, there were 27 fatalities amongst children aged 0–7 years (1.004 per 100,000) in Australia [1, 2]. The rate at which children are seriously injured is approximately four times greater at 4.34 per 100,000 children aged 0–4 years [2, 3].

Aboriginal and Torres Strait Islander people comprise 3% of the Australian population, and a significant proportion live in major cities (33%; 233,100 people) [4]. The suburbs of greater Sydney are home to the highest population of Aboriginal people of any Australian city (10%; 54,746 people) [5]. Fatalities and injuries are disproportionately higher in the Aboriginal and Torres Strait Islander population, where child passengers aged 0–4 years suffer 4 times more fatalities and 2.2 times more serious injuries than non-Indigenous Australians [6].

Child car seats are a proven, effective intervention that can reduce the incidence of serious injury and death amongst child passengers in motor vehicle crashes [7–9]. Using a car seat correctly is critical to a restraint system’s performance in the event of a crash [10]. Despite this evidence, the rate of use of appropriate child car seats in Australia has been historically low [11, 12]. Further, while there are no published estimates of the use of child car seats in the Aboriginal population, reports from crash data suggest usage rates are low [6].

The Australian government revised the national child restraint laws in 2009 and enacted these in New South Wales in March 2010 [13] requiring children up to 6 months old to be restrained in a rearward-facing child car seat, then a forward-facing child car seat until the age of 4 and a booster seat from 4 to 7 years old. It is expected that these revised laws, in combination with enforcement campaigns, will increase child car seat use [14, 15] and that education, such as hands-on instruction on car seat use or disbursement of car seats, will further increase consistent and correct use [16, 17]. However, mainstream community wide educational campaigns have been shown to be less effective when provided to minority groups where use of adult seat belts is less frequent and different barriers to car seat use are likely to exist [18]. In the USA, child car seat programs have been developed for American Indian/Alaskan Native children which address cultural needs in these communities with relative success [19]. Similarly, targeted programs are needed to promote use of child car seats in Aboriginal communities in Australia.

Aboriginal Community Controlled Health Services (ACCHS) are autonomous services governed and driven by the local Aboriginal community to deliver comprehensive and culturally appropriate health care. The model that underpins these services is founded upon research, evaluation and planning to provide effective community engagement, clinical services, health promotion, and cultural safety and awareness [20, 21]. ACCHS are the largest private employer of Aboriginal and Torres Strait Islander people within Australia, with a total estimated workforce of 5829 people, 55% of which are Aboriginal and Torres Strait Islander [22]. ACCHS services are appropriate locations for delivery of this program considering the record of success that these types of services have achieved in improving access through removing unintentional racism and breaking down preventative barriers to health care [23].

There have been a number of studies evaluating interventions to promote the use of appropriate child car seats. The 2006 Cochrane review by Ehiri et al. reviewed interventions to promote booster seat use in 4–8-year-olds [24]. There were five randomised controlled trials, including one in Australia by Bowman in 1987. The meta-analysis found a positive effect for interventions which promoted use of booster seats in 4–8-year-olds (relative risk (RR) 1.45, 95% CI 1.05–1.96). Greater effects were found when education and distribution of booster seats were combined (RR 2.34, 95% CI 1.50–3.63) [25]. A review which included younger children found strong evidence supporting legislation, education and the distribution program, noting also that there was no evidence of benefit from programs which relied on education alone [14].

Prior to this formative evaluation, the *Buckle-Up Safely* program was developed by the investigative team as a pre-school-based program and evaluated in a cluster randomised trial in South-Western Sydney (ranked in the lowest 30% of local government areas in Metropolitan Sydney by the Socio-economic Indices for Areas (SEIFA)) [17] and later in three pre-schools in regional New South Wales where the proportion of the Aboriginal population is more than twice as large as the national average (6.2% and 2.6%, respectively) families [26, 27]. These local studies provided further evidence that an integrated education, distribution and car seat fitting program improved the use of correct child car seats beyond that achieved by legislation alone. Based on these recommendations, and within a theoretical framework for planned behaviour, the *Buckle-Up Safely* program sought to include the key components of hands-on demonstration and car seat subsidies as well as delivering a clear and consistent educational message.

The aims for this study were (1) to identify the barriers to appropriate child car seat use amongst urban Aboriginal children to develop and implement a multi-faceted program and (2) to conduct an evaluation of the feasibility, in terms of ease of integration into the service and acceptability of this program in the setting of an ACCHS.

## Methods

Campbelltown and Mount Druitt are within greater urban Sydney and represent regions within NSW with higher concentrations of Aboriginal and/or Torres Strait Islander people [4]. Both regions rank within the lowest quartile of local government areas in Australia by SEIFA (21 and 12%, respectively) [28]. It is for this reason that both regions were selected for the delivery of the *Buckle-Up Safely* program.

The Aboriginal Medical Service Western Sydney (AMS WS) was located in Mount Druitt—approximately 40 km west of the Sydney Central Business District (CBD), within the Deerubbin Local Aboriginal Land Council Area. Tharawal Aboriginal Corporation is located in Airds, 56 km south-west of the Sydney CBD. Both organisations are ACCHS.

**Fig. 1:**
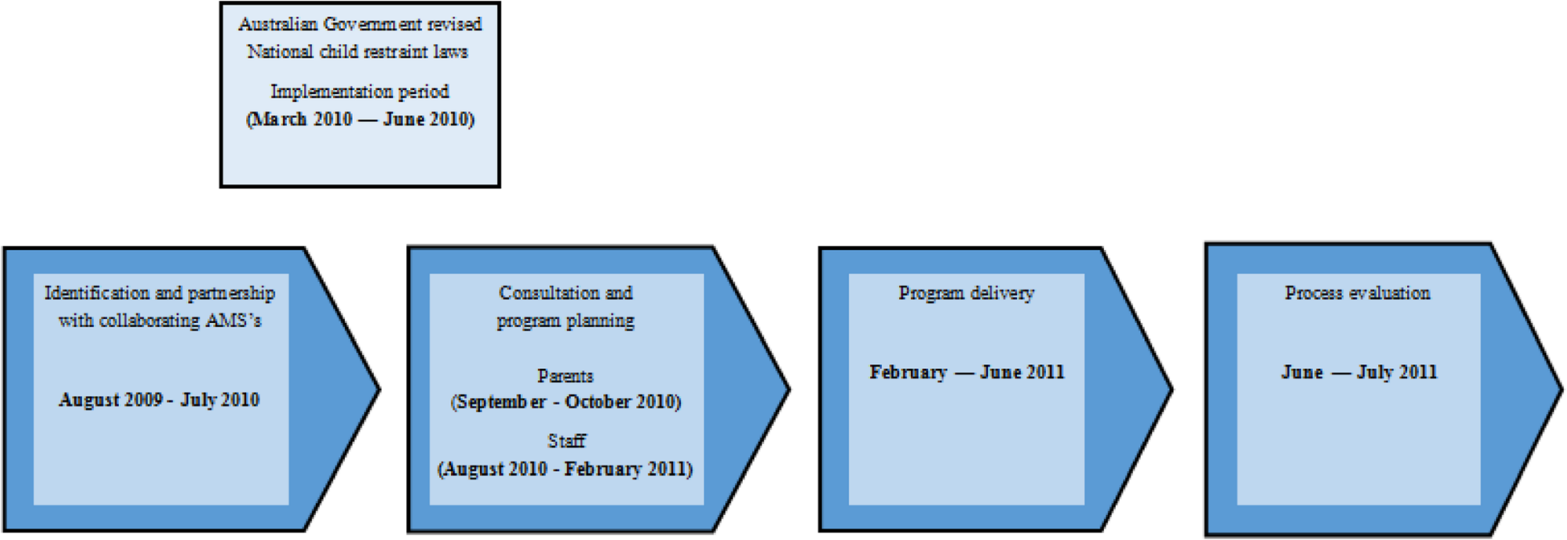
Program timeline

Consultation and program planning commenced in 2010 when the revised restraint laws were enacted in New South Wales [13], and the program was delivered in 2011. The timeline for the program can be viewed in Fig. 1.

Qualitative research was appropriate as we were seeking to explore behaviour and processes for implementation of a child car seat program in a specific setting. For the consultation with families and carers, and staff of the ACCHS’s research, staff used focus groups and in-depth interviews. Both are well-established qualitative methodologies, which have been used to explore barriers to child car seat use in other settings [29]. These methodologies are suitable when groups of people are likely to have common concerns or shared experiences [30].

The transcriptions were coded in NVIVO software (Version 9, QSR International) [31] within a framework of analytic categories of Barriers and Facilitating Factors for each stage of the adapted Precaution Adoption Process Model (PAPM). The PAPM has previously been adapted for other injury prevention practices [32]. The PAPM [32] was appropriate for the framework for analysis of the focus group transcripts as it recognises the stages of behaviour change. The seven stages in this model include being ‘unaware of the issue’, ‘aware of the issue but not personally engaged’, ‘engaged and deciding what to do’, ‘planning to act but not yet having acted’, ‘having decided not to act’, ‘acting’ and ‘maintenance’. These stages align with the process of achieving best practice child car seat use, which involves not only an understanding of the safety benefits but also purchasing an appropriate seat, installation and correct use for all journeys. The PAPM stages were simplified as a framework for this thematic analysis into ‘awareness’, ‘engagement’, ‘getting the right child car seat’ and ‘using correctly’. Thematic analysis is a process where patterned meaning is surmised across a dataset. Themes and sub-themes were identified, coded and categorised. Thematic analysis was used to synthesise the opinions, attitudes and beliefs expressed by the participants. The findings were used to inform the educational and child car seat distribution components of the program.

### Parents and carers

A series of three focus groups were conducted at the ACCHS involving parents, expectant mothers and grandparents of pre-school children. The focus groups were as follows: AMS WS on 8 September 2010 attended by 3 men and 9 women and Tharawal 8 September 2010 attended by 3 women from the Indigenous Childrens Program, and then on 26 October 2010: 13 women from the Art Group. The focus group sessions, facilitated by one of the researchers (LK) and observed by one of the investigative team (ML or KH), covered the following topics: (1) attitudes and knowledge people have about how children should travel in cars, (2) influences on decisions about how children should travel in cars, and (3) resources, information access and needs. At the end of the focus group, a summary of the discussion was presented and the opportunity given for further input and confirmation.

### Staff of the ACCHS

ACCHS staff were selected by research staff as ideal members of both communities to deliver the program. Their role embedded within the ACCHS and their pre-established relationships particularly with the targeted families gave them close insight into the nature of day to day interactions with families. Researchers met with the ACCHS staff on four occasions from October 2010 to February 2011 for an in-depth discussion to interview and discuss the program to identify ways that the existing program could be tailored to these two ACCHS sites. The intention of these consultations was to give staff members an opportunity to offer their knowledge of child car seat use within the community, to express what suggestions they had regarding the delivery of the program and also to advise on the program structure. The format of these consultations included planning meetings (Tharawal: 26 October 2010 and AMS WS: 28 February 2011), staff workshops (Tharawal: 14 November 2010) and presentations at staff meetings (AMS WS: 6 December 2010).

### Program development

The *Buckle-Up Safely* program was designed to (1) increase knowledge and engagement with the community on the issue of safe travel in cars, (2) provide access to subsidised child car seats and (3) train staff at the services to be able to check car seat installation for clients. Results from the focus group discussions and consultations with staff were used to tailor the program through its means of delivery so that it was designed to address the identified barriers and facilitators in this setting.

Child car seats were supplied to families at a subsidised cost. Staff at both services were provided with a bound manual including a script and the DVD inset in the cover. Individual training was provided to demonstrate how the manual could be used; however, the staff preferred the research team to facilitate these sessions as they were not confident with the content and the amount of information in the early stages of the program. An interactive session was designed including scripted responses to frequently asked client questions.

Further staff training opportunities included a specialised 2-h workshop ‘Out and About with Kids in Cars’ delivered by *Kids and Traffic Early Childhood Road Safety Education Consultants* (hosted on 19 April 2010 at Tharawal Ooranga Wandarrah Childcare Centre with seven attendees) and a full-day workshop on ‘Selection and installation of Child Restraints’ delivered by *Mobility Engineering* (hosted on 19 June 2011 at Tharawal attended by 14 staff).

### Evaluation: staff of the ACCHS

Semi-structured interviews were completed with 13 staff within 4 months of program completion. The evaluation included nine staff from Tharawal Aboriginal Corporation and four from AMS Western Sydney encompassing program coordinators, program advisors, the supervisor of Ooranga Wandarrah Preschool at Tharawal, nurses, administrators involved in car seat distribution, health education officers, clinic coordinators and case workers.

The purpose of this process was to gain insight into the acceptability of the program and to understand what elements worked well and what could be learnt from the delivery of the program.

The research study was approved by the Human Research Ethics Committees at the University of Sydney (reference #12807) and the Aboriginal Health and Medical Research Council of New South Wales (reference # 732/10).

## Results

### Identifying facilitators and barriers—parent focus group discussions

The themes are described below in relation to the adapted child car seat model of behaviour change and are summarised in Table 1. The purpose of tabulating these results was to identify what barriers and facilitators exist at each stage of transitioning families through the adapted model of behaviour change to what the program can focus upon in its delivery.

**Table 1:** Issues surrounding use of child car seats grouped into barriers and facilitating factors for the stages of behaviour change

Stages	Barriers	Facilitating factors
Awareness	Lack of awareness of the law and confusion about the age and seat types required ‘No ideas, haven’t heard’ and confusion about the age and seat types required. ‘Most people don’t know, like my sister thinks only if they are little -like toddlers - once they hit school she thinks it is the right thing.’	Having an understanding of exactly what happens in a crash Having visible pictures/posters to inform
Engaged	Adults do not think about safety on day to day journeys ‘Some just don’t even care - not worried. Just get in the car’ Conceding for short trips ‘What do I do, I am only going down the road…’ ‘if you drove around all day thinking about crash, you won’t get in the car’	Involving children in safety: doing workshops with children to educate children about why car seats are important. Kids can see better—sitting up high ‘I say it is his ‘special seat’ Involving peers in safety benefits: buy a seat for use with children, you drive in cars that are not your own and loan to peers when required. set example to peers and sharing a story of someone who has had a child injured in a crash (visual)
Getting the right restraint	Confusion: large children concern that large children do not fit in the seat they need by law. ‘Eyes over or head over. What do I do?’ Confusion over age, weight and height guidelines for selecting the right seat	Individual advice **‘**they need to show people if your kids are bigger, then you should get a different car seat, should be a bit wider. Just get a different seat Good product range and advice ‘The booster ones - some can be really big, with the seatbelt. They are not all small’ Height markings so you know when they have grown out of the seat
	Lack of resources/cost: do not own car seats, cost was a major barrier, especially with more children and currently having no seats, cost of restraint fitters also acknowledged. ‘paying a ridiculous amount for just a foam chair’	Supportive of hire scheme or subsidised purchase
	Large families: problems fitting seats across back seat and doing seat belt up with tight fitting seats New baby need the car seat, so we need to move the toddler on to a booster seat before he technically should. ‘I want him in a seat before the next baby comes’	Appeal of seats that will last across greater age range
		Simple tick system favoured as a way to get better seat. ‘As long as you have the tick, cheapest one with that tick on it. Means I am safe and abiding by the rules.’
Using correctly	Multiple vehicles: taxis not having seats, and having to have car seats to put in other people’s cars, difficult to move between cars. ‘Taking in and out, in and out. It’s such a headache’	Ease of use: group felt that once familiar, seats are easy enough to get in and out of a car
	Children resistant to using child restraints: children do not like to sit in ‘baby’ car seats, seats not comfortable Other carers not understanding need for child restraints Unplanned passengers: while participants knew children needed to be in restraints, they were conflicted when asked to carry extra passengers ‘you might be going to pick somebody up and they might bring 2 kids out to the car and you only have one seat. What do I do?’	Being consistent with children—if you are not in the seat, we do not drive anywhere. Making sure other adults who may take the kids in their car have the same rules. Being consistent with peers, child cannot travel without restraint, own seats for your vehicle and loan out. ‘I made her pull over and I just started walking, because she didn’t do it. ‘ Threat of police enforcement: ‘Not much you can do without getting in trouble’ ‘Can’t afford a fine’
	Fitting challenges: cars without the right anchor points—vans, problems with two door cars	Restraint checks are useful: understood importance of restraint check, knew about fitting checks
		Demonstration favoured getting a practical demonstration and thought it should be at point of sale/supply. ‘Demonstration would be best, better than reading a book. You pay attention to someone actually standing there’

#### Stage 1: awareness

Not all participants were aware of the new legislation. Amongst those who were aware, there was confusion about the appropriate car seat for age and when to progress children to the next car seat. These findings reinforced the need for the program to increase awareness and include education about child car seat use.

#### Stage 2: Engagement

When focus group participants were asked to review program materials, they reported a preference for simple instructions with pictures. Participants also felt that the best approach to engaging parents was to just ‘have a yarn^1^’ about keeping kids safe in the car, and this was reinforced by beliefs that individual, practical advice and demonstrations of car seats were more important than brochures. Additionally, participants reported that the educational DVD would not be watched if given out. While participants were generally supportive of the law, some participants reported frustration at governments making changes to the law without helping people keep up with them.

#### Stage 3: getting the right car seat

Participants understood the risk of injuries in road crashes and reported that transport of children was a shared responsibility across community. It was also identified that transporting additional children posed a challenge to carers who were trying to use car seats correctly and that they often felt pressured to travel with extra children whether or not they had the right car seat.

Obtaining a car seat was influenced by safety and cost considerations. Logistical issues discussed included the needs of large families—fitting multiple car seats in a car, but also, these families found it difficult to hand down car seats from child to child while ensuring each child remained in the right seat for their age. Participants felt that while ‘nothing in life is free’, they thought that subsidised seats should be available and suggested 50% of the retail cost was most suitable.

#### Stage 4: using correctly

Though there was interest in further information about correct use of car seats there was also a strong feeling of self-sufficiency: ‘work it out for yourself because there is nothing there to say if your kid doesn’t fit into this at this age, then do this’. There were also reports of children being excluded from trips due to inability to comply with child restraint law.

### Identifying facilitators and barriers—staff interviews and discussions

The staff acknowledged that their daily work provides opportunities to teach clients about appropriate use of child car seats. Further, staff at both services explained that they had been active in checking and securing children during transport in their work. All agreed that there is a role for them to understand the new laws and to pass this information on to families.

Staff stated that although they had been exposed to information regarding the revised laws recently and had a reasonable understanding of the safety benefits of age appropriate car seats, there was still confusion over the legal age at which a child can travel in the front seat and the transition ages for different types of car seats.

Staff acknowledged the challenges of engaging with families on the issues of child car seats and relayed that there had been limited uptake in previous initiatives. They offered suggestions including providing programs that focus directly upon children, such as toddler groups; using posters and flyers; hosting discussion groups, information sessions, and one-on-one discussions; involving doctors; screening a DVD on a loop; identifying specific parents and following up on information provided. The staff felt that information should be concise, clear and easy to understand as clients are unlikely to read lengthy resources and should be accompanied by a ‘hands-on’ demonstration.

### Developing and implementing the program

The program components supporting each of the stages of behaviour change are summarised in Table 2. To raise awareness, the *Buckle-Up Safely* program ACCHS health workers were encouraged to focus upon spending time engaging in personal and individual client interactions ‘yarning’ with families to communicate the core messages of safe travel for children. This would be later followed up in incidental conversations.

**Table 2:** Program content and corresponding components of the simplified precaution adoption process model

Stages	Program content
Awareness	• Personal approach to clients • Posters, printed material • Policy on transport of clients
Engaged	• Presentation at community days and information sessions • Presentation at staff training and workshops • Interactive material (height charts)
Getting the right restraint	• Demonstration of seats to families • Selection of a forward facing child restraint and a belt positioning booster seat based on the Child Restraint Evaluation Program (https://www.childcarseats.com.au/) • Supply child restraints and booster seats at subsidised cost of $50 to client
Using correctly	• Staff trained to be able to install the child restraints correctly • All staff familiar with appropriate child restraint for age • Integrated themes of correct use and right seat for age into parenting programs

In response to the identified barriers and facilitators identified in the focus groups and in-depth discussions, resources were chosen to ensure high pictorial and interactive content while conveying clear simple messages about child car seat use and the new law. The resources available included a DVD explaining how child car seats protected children from injury, print material, a height chart and different types of child car seats for demonstration purposes (http://www.thegeorgeinstitute.org/videos/buckle-up-child-car-restraints) [17, 33]. Training resources available through the New South Wales State Government’s road agency were also utilised. These resources were distributed to families through the one-on-one interactions with families, were made available to families in the foyer of each ACCHS, and distributed in bags at the community event.

As parents and carers both acknowledged that the transportation of children was a shared responsibility of the community, educational content and advice was delivered at a ‘Close the Gap Community Day’ information stall in collaboration with the Aboriginal Program Officer at the Roads and Traffic Authority (Tharawal—24 March 2011). An information session was also co-presented to the local community by the project team and staff (Tharawal—25 March 2011). No family sessions were held at AMS WS.

As a response to the cost of car seats being a restrictive barrier, staff and families agreed that having seats available at a subsidised amount rather than free of charge was preferable to ensure parents valued the purchase. As such, an amount of $50 was agreed upon (full retail price $150–$300), though some families had difficulty meeting the payment. A coordinator was appointed to manage orders and followed guidelines to deliver age- and size-appropriate car seats. The program made up to 30 child car seats available to each service. Thirty-three families placed orders for 21 seats (8 booster and 13 forward facing seats) at Tharawal and 12 seats (2 booster and 10 forward facing seats) at AMS Western Sydney.

Two seats were provided to each service at no cost for use during demonstrations to clients. In addition, AMS WS received two seats for loan purposes to clients who were making ad hoc trips to regional areas. While having an authorised restraint fitter available on site was considered, staff reported that from previous experience these fitting days had low patronage.

### Staff evaluation of program feasibility and acceptability

All respondents involved in client outreach programs indicated that communicating about safe travel in cars was relevant to their role at the health service and agreed that this was as important as other health issues.

Staff responded that the best aspects of the educational content were (1) clear information, (2) practical demonstration, and (3) clear representation of the consequences of children being unrestrained or inappropriately restrained in a car crash. Staff reported that transporting children is a regular aspect of daily operations for health checks and participation in other programs and activities.

The 145 cm height chart was particularly popular in engaging families to think about the size of their children and how well they fit an adult seat belt. There was enthusiastic support for the practical training in car seat selection and fitting. Seven of nine staff responded that they either agreed or strongly agreed that they now had a clear understanding of the laws. At one ACCHS, training at the general staff meeting was instrumental in establishing a uniform level of understanding of child car seat use where there were previous disagreements. Having an external advisor present was reported to help resolve and clarify these issues—this indicates the benefits of supporting program staff during the early stages of program implementation to feel confident and well informed. Further, staff at both services recommended having a person dedicated to the program implementation and that this would enhance the program fidelity.

## Discussion

This pragmatic study has shown that a child car seat program delivered through ACCHS, informed by community consultation consisting of access to information, hands-on demonstrations, subsidised car seats and car seat checks performed by appropriately trained staff was feasible and accepted by the local staff of the ACCHS in Western and South-Western Sydney. Further, this study has shown that within both communities there exists a strong demand for subsidised child car seats to enable car seat use.

A large part of this program was dedicated to building capacity within the ACCHS, and it has been the increased understanding of staff, their input and advocacy for the program, which has contributed to the program’s legitimacy within community thus strengthening the program beyond what could have been achieved by research staff within the given timeframe.

Staff interviews highlighted that they felt there is a role for them to understand and pass on information about child car seat laws to families. Despite having been exposed to information regarding the revised laws recently, there remained some uncertainty about the law’s particular details. As Kakefuda et al. have found, health care workers (like the community) often do not realise that motor vehicle crashes are a major cause of death and serious injury in children [34].

Like the staff, many clients noted that they were somewhat confused or had little awareness of the changes made to child restraint legislation. Qualitative research in another population groups has found that a lack of parental knowledge of benefits, differences in risk perception and ‘negotiability’ in parenting style can be barriers to increasing correct car seat use [29]. The *Buckle-Up Safely* program found similar issues.

The clients and staff both conveyed that one-on-one demonstrations would be most effective and that the best means to communicate with families would be to have a ‘yarn’ rather than formal presentations. This approach was adopted, and text-heavy brochures were avoided. As community members felt that the safe transportation of children was the shared responsibility of the community, the program was delivered at a community event. In this community, large families and needing to pick up additional children posed a barrier to safely transporting children each trip. Further financial barriers were documented whereby families may not be able to afford the cost of a new child car seat to ensure that their child is in the right type for their age. These barriers and facilitators identified by staff, and family and carers represent the distinct characteristics of the Western and South Western Sydney Aboriginal community. Others have predicted concurrent barriers to child car seat use amongst Aboriginal communities, specifically the low rate of adult seat belt use, use of multiple vehicles, multiple carers being involved in transporting children and over-crowding of vehicles [35–37].

It is important that health services are sensitive to, and acknowledge and respect, cultural factors and similarly are wary of potential physical and/or economic barriers that can prevent families from accessing and learning from the available health service [38]. Understanding these barriers and facilitators unique to the community in which the service is operating is the first step towards developing a successful health program.

Child car seats have been part of several previous road safety initiatives targeting Aboriginal people in Australia. The Aboriginal Seat Belt Campaign in South Australia provided information on child car seats in the form of brochures and stickers featuring Aboriginal artworks and illustrations to facilitate the safety message to the community [39]. Child car seat promotion was also included in a mass media campaign on Indigenous Road Safety in the Kimberley [39]. This program is the first reported evaluation of a multi-faceted health promotion program directly targeted at child car seat use delivered via community health care workers of ACCHS. This program has also been unique in its community consultation for the delivery.

Understanding and harnessing the barriers and facilitators to appropriate car seat use has contributed to the degree to which the *Buckle-Up Safely* program has been able to tailor and navigate community factors [36–38]. This has been a key to the suitability and acceptability of the program.

## Conclusions

In summary, a program was developed to promote child car seat use in urban ACCHS, which was well supported by clients and staff. The program focused on developing capacity amongst staff, made use of existing infrastructure and adapted resources for use in this setting. It is clear that locally developed and owned programs are needed for Aboriginal communities. The program developed from this formative evaluation, with consideration of the barriers and facilitators to child car seat use, has shown promise, but will need to be tested in a larger trial.

## Abbreviations

ACCHS: Aboriginal Community Controlled Health Services
AMS WS: Aboriginal medical service Western Sydney
PAPM: Precaution adoption process model
SEIFA: Social-economic Indices for Areas

## Footnotes

1 This is a colloquial term for having an informal discussion, often used in Aboriginal communities
